# Boosting forward-time population genetic simulators through genotype compression

**DOI:** 10.1186/1471-2105-14-192

**Published:** 2013-06-14

**Authors:** Troy Ruths, Luay Nakhleh

**Affiliations:** 1 Department of Computer Science, Rice University, Houston, USA

## Abstract

**Background:**

Forward-time population genetic simulations play a central role in deriving and testing evolutionary hypotheses. Such simulations may be data-intensive, depending on the settings to the various parameters controlling them. In particular, for certain settings, the data footprint may quickly exceed the memory of a single compute node.

**Results:**

We develop a novel and general method for addressing the memory issue inherent in forward-time simulations by compressing and decompressing, in real-time, active and ancestral genotypes, while carefully accounting for the time overhead. We propose a general graph data structure for compressing the genotype space explored during a simulation run, along with efficient algorithms for constructing and updating compressed genotypes which support both mutation and recombination. We tested the performance of our method in very large-scale simulations. Results show that our method not only scales well, but that it also overcomes memory issues that would cripple existing tools.

**Conclusions:**

As evolutionary analyses are being increasingly performed on genomes, pathways, and networks, particularly in the era of systems biology, scaling population genetic simulators to handle large-scale simulations is crucial. We believe our method offers a significant step in that direction. Further, the techniques we provide are generic and can be integrated with existing population genetic simulators to boost their performance in terms of memory usage.

## Background

Forward-time population genetic simulators are critical research tools in evolutionary biology, as demonstrated by both the growing number of available simulators and the collection of high-impact studies that employ them [1]. These simulators allow for in-silico generation and testing of evolutionary hypotheses that would otherwise be intractable to generate or test in a laboratory setting due in large part to the nature of the process. Evolution “is a loose and complex process, the result of a number of interacting, individually weak forces with many alternative outcomes” [2]. Consequently, forward-time simulators are ideal for tinkering with these weak forces—changing the ones that are modeled and their relative strengths or rates—in order to observe the many alternative evolutionary outcomes. Unlike backward, or coalescent, approaches to evolutionary analysis, forward-time simulators can handle the growing bevy of known evolutionary processes and environments [3].

But forward-time simulations have their limitations: a critical design pivot exists around execution speed, memory usage, and flexibility [1]. Available simulators necessitate a trade-off between flexibility and speed for realistic simulations to be feasible, and often require the user to adjust the evolutionary scenario to fit the capability of a certain simulator using scaling factors. This results in a large collection of simulators that require a decision flowchart to choose an appropriate simulator [4]. For example, methods were recently developed to increase the execution speed of simulations; however, these gains in speed come at the expense of reduced flexibility [5]. Because forward-time simulations track complete ancestral information, including all alleles which arose but were lost, the imposed computational burden limits the potential scope of the problem [3]. Even leveraging rescaling techniques that they employ, such as altering the input parameters to diminish the population size and number of generations, to improve computational efficiency does not evade this critical issue of computation time and memory usage. Simulating large sequences on the order of 10 Mb “tends to make forward simulators crash due to memory usage” [3], which is compounded by the stochastic and unpredictable nature of these simulations. Further, more complex genotypes, such as protein and RNA structures, regulatory pathways, and epigenetic mechanisms, are studied using forward time simulators [6,7]. Although current simulators exist for efficiently simulating large genomic regions — FREGENE, SimuPOP, or GenomePop — the memory management techniques do not extend to arbitrary genotype representations like pathways or metabolic networks or other mutation types like insertions or rearrangements [8,9]. For instance, SimuPop provides a compression module which efficiently encodes long sequence regions with rare mutant variants [10]. In addition, general lossless data compression algorithms cannot scale to forward-time simulator scenarios where very large (>100 MB) data strings must be compressed and decompressed thousands of times per generation for thousands or millions of generations. Compression and decompression that require on the scale of minutes — as is the case for general lossless compression algorithms — is completely infeasible as a general solution. Ultimately, the constraint on memory is a major roadblock to the application of forward-time simulators to both complex biological structures and processes and large problem scopes.

In this work, we develop novel methods for addressing the memory issue inherent in forward time simulations by compressing, in real-time, active and ancestral genotypes. We propose algorithms which can be implemented in any current simulator and are independent of the evolutionary model (our algorithms work for both the Moran and Wright-Fisher models). Specifically, our contributions are two-fold: the operation graph, a compression data structure for forward-time simulators, and Greedy-Load, an algorithm for improving the decompression performance of the operation graph by managing a strategic cache. The algorithms we present work equally to compress the whole ancestral information or just the active alleles of a simulation. Compressing the ancestral information of extant genotypes retains important information that would otherwise be lost without our compression algorithm.

Computer simulations have long played a central role in deriving and testing hypotheses in evolutionary biology and population genetics. Thus far, population genetic simulations have for the most part employed either abstract genotype constructs or very short sequences. As biology ushered in the post-genomic era, and more specifically the systems biology era, understanding whole systems and organisms has leaped to the forefront of research. The availability of data from different species, and even different individuals in a population, has led to efforts to incorporate evolutionary analyses in systems biology [11] and synthetic biology [12]. To scale population genetic simulators to this new era, where genotypes can encompass an entire genome, interactome, or organism, it is imperative to address the computational requirements of existing simulators so as to enable handling such large-scale genotypes and flexible genotype representations. Our work offers a significant step in this direction. Further, our methods are generic so as to allow integration with other existing, popular population genetic simulators.

## Methods

In this work, we propose a real-time compression algorithm for reducing the memory footprint of a forward-time population genetic simulation, composed of two components: the compression technique (operation graph) and the decompression accelerator (Greedy-Load). The operation graph represents each genotype by the sequence of evolutionary events that gave rise to it, and Greedy-Load maintains a “small” set of explicit genotypes that accelerates the decompression of compressed genotypes in the operation graph. Whenever the simulation or analysis requires access to the genotype information, genotypes can be retrieved on-the-fly by applying the evolutionary events to an explicitly represented genotype. We now describe the algorithm and data structure we use in detail, including the decision on which genotypes to represent explicitly, how to decompress a genotype, and how to build/augment the compression data structure. We begin with the compression technique, which we call the operation graph.

### The operation graph

As evolutionary operations — such as mutation or recombination — occur in the population genetic simulation, the dependency of each operation on the previous genetic history is encoded in the operation graph (OG). Operations are stored as nodes in the OG, a directed acyclic graph (DAG) structure, where operations with one incoming edge correspond to mutations and with two incoming edges correspond to recombinations. Each operation that arises over the course of the simulation is encoded as a distinct node in the OG, along with the genetic material produced by the operation.

Let F denote the set of evolutionary operations allowable in a simulation, and let G denote the set of genotypes that arise during a simulation. For mutational evolutionary events, each element op∈F is a function op:G×Φ→G, where op(A,ϕ)=C denotes that genotype *C* is the result of applying evolutionary event *op* to genotype *A* with parameters *ϕ*. However, for recombination, op∈F is a function op:G×G×Φ→G, where *op*(*A*,*B*,*ϕ*)=*C* denotes that *C* is the result of a recombination event involving genotypes *A* and *B*, with parameters *ϕ*.

For example, if we take ϕ=〈base-pair- mutation,3,T 〉 and apply it to genotype A=ACCAAAT, we obtain genotype C=ACTAAAT, since the operation applied to *A* is a base-pair mutation that substitutes nucleotide *T* in the third position. Since different evolutionary events have different types of parameters, in addition to the “input” genotypes *A* and *B*, we abuse notation, for the sake of simplicity, and use *op* as a function from G to G for mutation and G×G to G for recombination—additional parameters *ϕ* for applying *op* should be clear from the context.

The operation graph (*OG*) is a rooted, labeled, weighted DAG *OG*=(*V*,*E*,*ℓ*,*f*,*w*,*c*), where 

1. *V* is the set of nodes;

2. *E*⊆*V*×*V* is the set of edges;

3. ℓ:V→(G∪{nil}) is the genotype labeling function with the constraint that {*v*∈*V*:*ℓ*(*v*)≠*nil*}≠*∅*;

4. f:V→F is the operation labeling function;

5. w:V→ℝ is the weight function such that *w*(*v*), for node *v*∈*V*, is the frequency of the genotype *ℓ*(*v*); and

6. c:V→ℝ is the cost function such that *c*(*v*), for node *v*∈*V*, is the non-negative computational cost of applying the operation *f*(*v*).

A node *v* is called explicit if *ℓ*(*v*)≠*nil*. That is, an explicit node corresponds to a genotype that is not compressed.

For a node *x*∈*V*, we denote by *Anc*(*x*)⊆*V* the set of all lowest explicit nodes between*x* and the root of *OG*, where a node *y* is lowest if no explicit node *z* (*z*≠*x* and *z*≠*y*) resides on a path between *y* and *x*. In particular, if *x* is explicit, then *Anc*(*x*)={*x*}. The set of active nodes in an OG, denoted by *A*(*OG*), is all nodes whose corresponding genotypes have non-zero frequency; that is, A(OG)={v∈V:w(v)≠0}.

#### Novelty of the operation graph

The OG is a compression technique similar to LZ77 with edit operations and uses a structure similar to the Ancestral Recombination Graph (ARG), a phylogenetic structure that describes the evolutionary history of a set of genetic samples [13-15]. The LZ77 algorithms replace repeated occurrences of data with references to a single copy of that data existing earlier in the input data stream. In our case, instead of repeated occurrences, we replace “evolutionary related occurrences”, such that we keep track of homologous, rather than identical, genotypes. For instance, if “ACCCT” evolved from “ACCGT”, only one instance is explicitly saved. Further, the operation graph is implicitly produced by forward time population genetic simulators, whether or not it is explicitly stored; whereas for LZ77, the identification of previous, similar strings is the bulk of the computational work in its implementation. Lastly, while LZ77 is a general compression scheme, the operation graph is biologically motivated, and in general, applies to scenarios where data evolves in a population, so that occurrences of data can be related to each other through evolution and this relatedness is used in the compression. For instance, it is not clear how LZ77 would handle the forking replacement dependencies incurred through processes like recombination.

While both the OG and ARG employ a DAG, the similarity between the two almost ends there. An ARG provides an explicit model of the evolution of a set of genetic sequences, mainly under point mutations and recombination [15]. The mutational model is often assumed to be the infinite sites, but more recent work has considered finite-site models as well [16]. On the contrary, the OG is an implicit representation of a set of related genetic information, where mutations and recombinations can be general (ranging from point mutations to insertions/deletions to genomic rearrangements). Further, while ARGs model the evolution of genetic sequences in a population setting, the OG is defined for arbitrary genotypes. A case in point is our recent population-level analysis of regulatory networks in E. coli, where the OG was defined over genotypes consisting of regulatory networks [17].

#### Updating the operation graph

Whenever a new genotype *C* arises from existing genotypes *A* and *B* through a recombination operation *op*, the operation graph is updated by (1) adding a new node *u* to *V*, (2) adding new edges *e*_1_=(*x*,*u*) and *e*_2_=(*y*,*u*) to *E*, where *x* and *y* are the nodes that correspond to genotypes *A* and *B*, respectively, and (3) setting *f*(*u*)=*op*. In terms of *ℓ*(*u*), it can be set to *nil* or to the new genotype *C*; we discuss below the choice we make in our algorithm. If the operation is a mutation, then only a single new edge is added in Step (2). The cost of *op*, or *c*(*u*), can be set based on the type of operation (*e.g.,* insertion, base mutation, deletion, recombination) or the input to the operation *ϕ*. In the case of recombination, the ordering of the two parents is handled at the implementation level.

Whenever a genotype *A* is lost from the population, the operation graph is updated only when when the node *x* that corresponds to genotype *A* is a leaf node in *OG*. In this case, the algorithm identifies the set *Y* where each node *y*∈*Y* is the lowest node on a path from the root to *x* that is either active, of out-degree 2, or the root of *OG*. Once node set *Y* is identified, allnodes on the path from *y*∈*Y* to *x*, excluding *y*, and all edges on that path, are deleted from *OG*. If *x* is not a leaf node, no update is done, since some active genotypes may be “under” it.

#### Measures of the operation graph quality

Given the graph *OG*, the genotype in every node can be decompressed; that is, for every node *x* with *ℓ*(*x*)=*nil*, the explicit value of *ℓ*(*x*) can be computed by traversing the path, or paths, from *x* to nodes in *Anc*(*x*) and applying the corresponding operations. The decompression cost for a given node *x*, denoted by *cost*(*x*), is

$$\text{cost}(x)=\underset{v}{\sum }c(v),$$

where the sum is taken over all nodes that resides on paths between nodes in *Anc*(*x*) and *x*. For a pair of nodes *x* and *y*, where *y* is on the path from a node in *Anc*(*x*) to *x*, we define the cost of decompressing node *x* by using information on the way from *y* to it, as $\text{cost}(x,y)=\underset{v}{\sum }c(v),$ where *v* ranges over all nodes on the path from *y* to *x* (*cost*(*x*,*y*)=0 if *y* is not on any path from a node in *Anc*(*x*) to *x*).

Further, the load of a node *x* (or, the corresponding genotype) is given by

$$\text{load}(x)\;=\;\underset{y\in U(x)}{\sum }\;w(y)\;\cdot \;\text{cost}(y,x),$$

where *U*(*x*) denotes the set of all nodes in *OG* that are under node *x* and require node *x* for decompression. Notice that for two operation graphs *OG*_1_ and *OG*_2_ whose underlying graphs are isomorphic and node labelings are identical, it may be the case that *cost*(*x*) based on *OG*_1_ is different from *cost*(*x*) based on *OG*_2_.

If we denote by C(V)={v∈V:ℓ(v)≠nil}, which is the set of uncompressed genotypes, then no compression is achieved when *C*(*V*)=*V*, and maximum compression is achieved when *C*(*v*)={*r*} for the root node *r* of graph *OG*. The time it takes to access the explicit genotypes is effectively the time it takes to decompress all the compressed genotypes.

### Compression algorithms

The set *c*(*v*) of an operation graph *OG* is at the core of the space-time trade-off here: the larger *c*(*v*), the more space is consumed and the less time is required to access the explicit genotypes, and the smaller *c*(*v*), the less space is consumed and the more time is required to access the explicit genotypes. Therefore, a central task here is to determine the set *c*(*v*) that would minimize the *load* of an operation graph. Here, we describe several compression algorithms for this task, one which is the main contribution of this paper — Greedy Load — and the others which are used for performance comparison.

#### Greedy-Load

In Greedy-Load, the inputs, in addition to the operation graph *OG*, are *k*, which is a pre-specified bound on the desirable size of *c*(*v*), and *t*, which is the number of generations elapsed between updates of the set *c*(*v*). This algorithm assumes that *load(x)* for all *x*∈*V* is implicitly calculated and updated whenever the membership of *c*(*v*) changes.

In a nutshell, Greedy-Load seeks to advance the set *c*(*v*) towards the leaves and active alleles of the *OG* by greedily caching genotypes with high levels of *load*. We define the utility function *advance(x)* which maximally “advances” the decompression from *x* towards the leaves of the *OG*: 

1. let node *y*∈*U*(*x*)∪{*x*} be the highest node that is either: 

(a) a leaf,

(a) has non-zero weight (frequency), or

(a) has at least two children each of which has non-zero load and is not in *C(V)*;

2. decompress the genotype corresponding to node *y* and set *ℓ*(*x*)=*nil*.

The Greedy-Load algorithm applies the following two steps on a given operation graph *OG* every *t* generations in the simulation (in the first application of this algorithm, we set *C*(*V*)={*r*}). In the first step, nodes that are no longer needed for decompression — *load*(*x*)=0 — are compressed, otherwise the decompression is advanced towards the leaves of the OG. In the second step, nodes are added to *C(V)* by decompressing the max-load child of the max-load cached node. 

1. For each node *x*∈*C*(*V*): 

(a) if *load*(*x*)=0 and |*C*(*V*)|>1, set *ℓ*(*x*)=*nil*, or

(a) if *load*(*x*)>0, perform *advance*(*x*).

2. Add nodes to *C(V)* until |*C*(*V*)|=*k* or no other nodes may be added. Let node *x*∈*C*(*V*) have maximum load in *C(V)* and node *y* be the max-load child of *x*, at each iteration 

(a) decompress the genotype corresponding to node *y*, and

(a) perform *advance(y)* and *advance(x)*.

##### Example execution

**Figure 1:**
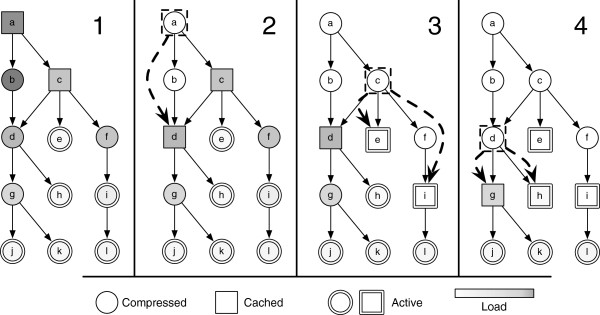
**Example execution of Greedy-Load.** An example execution of Greedy-Load is illustrated on an abstract operation graph. Each node *a-l* represents a distinct genotype (or allele) and each edge depicts evolutionary descent by mutation (one parent) or recombination (two parents). The actual genotype representation could be a sequence or pathway. Genotypes may be compressed (○), cached (□) or active (=). The *load* of each genotype is depicted as the background color, with darker colors corresponding to greater load. Sequential steps taken by the Greedy-Load algorithm are illustrated from left to write, showing the incremental changes that update the set of uncompressed genotypes from {*a*,*c*} in Panel 1 to {*e*,*g*,*h*,*i*} in Panel 4. Dashed arrows within each step illustrate which genotypes are compressed and uncompressed. For instance, in Panel 2, *d* is uncompressed (cached) and *a* is compressed. For this example, the total number of cached genotypes *k* is 4. A complete description of this execution can be found in the *Example execution* section in the Methods.

Assume an *OG* as illustrated in Figure 1, composed of 12 operations labeled *a* to *l* connected by 12 edges. Node *a* is the root and nodes *j,k,h,e,* and *l* are leaves. All nodes are mutation operations except for *d*, which is a recombination operation with inputs *b* and *c*.

Panel 1 in Figure 1 depicts the *OG* prior to the execution of Greedy-Load. All leaves correspond to genotypes that are active in the population in addition to the internal node *i*. This example walks through the application of Greedy Load with *k*=4.

In Panel 2, the first step of Greedy-Load ‘advances’ the decompression from *a* towards the leaves. In this case, node *d* has two children, *g* and *h*, each of which has non-zero load and is compressed. Because node *c* does not require *a* for decompression, it is not in the set of nodes considered in *advance(a)*. Because node *c* has two compressed children with non-zero load, it is not possible to advance the decompression from *c* towards the leaves, so nothing is done.

In Panel 3, assume load(c)>load(d) and load(f)>load(e), so *f* is decompressed and *advance(f)* is performed, which results in decompressing *i*. Because *i* corresponds to a genotype that is active in the population, *i* may generate decompression requests, and so decompression cannot progress down the *OG*. In addition to *advance(f)*, *advance(c)* is also performed, which results in the decompression of *e* because *c* has only one child with non-zero load.

In Panel 4, because C(V)<4 and load(d)>load(i), node *g* is decompressed and *advance(g)* and *advance(d)* are performed. Because *g* has two compressed children with non-zero load, decompression cannot be advanced further down the *OG*; however, because *d* only has one compressed child with non-zero load (since *g* is now decompressed), then *d* is compressed and *h* is decompressed. At this point, C(V)={e,g,h,i} and the application of Greedy-Load is complete.

In more realistic simulation scenarios, the *OG* is both much wider and taller than presented in this simple example execution, so we visualized the execution of Greedy-Load on more complicated *OG* topologies (see Additional file 1). In this animation, the evolution of the *OG* is visualized along with the set *C(V)* for scenarios with low and high recombination rates.

#### Other compression algorithms

In order to measure the performance of Greedy-Load, we defined two additional compression policies that make fast, but potentially poor (in terms of memory and execution speed), explicit representation decisions. Unlike Greedy-Load, these simple comparison compression algorithms or policies do not require knowledge of the entire operation graph to select the explicitly stored genotypes. Current simulators store active genotypes that arise during the course of a simulation; we refer to this policy as Store-Active. The alternative is to store only the root genotype(s) in the operation graph, which we call Store-Root. More formally, for an operation graph *OG*=(*V*,*E*,*ℓ*,*w*), we have: 

•Store-Active: set *C*(*V*)=*A*(*OG*).

•Store-Root: set *C*(*V*)={*r*:*r* is a root node in *OG*}.

### Implementation

We implemented a population genetic simulator and the compression algorithms in C++, which can be used as a development library or a command line tool. It is important to note that we used explicit memory management, rather than garbage collection, for genotype data structures, so memory usage metrics are honest measurements of allocated memory. The emphasis in this work is on the compression algorithm rather than the implementation of a memory-bounded forward-time population genetic simulator. We did not find any existing simulator with a software architecture that allows for integrating (without completely overhauling the implementation) a memory management policy, such as the ones we propose here: hence, our choice to implement our algorithms independently of existing simulators. However, we still provide a command line tool which, in addition to taking flexible input parameters, provides an example for how the compression techniques in this paper may be integrated into a pre-existing simulator.

To improve the performance of the population genetic simulation with a memory-managed genotype heap, we implemented both partial and batch decompression. In partial decompression, rather than uncompressing a 100,000 bp sequence to access only 10 bp, we implemented intelligent decompression which could retrieve randomly accessed locations without decompressing the entire sequence. Because each operation in the *OG* stores meta-data associated with its application (such as locations and mutations), we implemented operations such that they can be applied on the entire sequence or on a given index. In batch decompression, we implemented the population genetic simulator such that it reduces the data requests of a particular genotype. For instance, during a mutation event involving multiple base-pair changes, the genotype is uncompressed once and used repeatedly rather than uncompressed with each base pair change.

Because calculating *load* on the OG may be a costly exponential calculation, we tracked the number of data requests per operation as a proxy for *load*. For all operations in the *OG*, the number of data requests are set initially to zero and increment during the population genetic simulation. The number of data requests increments by one when the population genetic simulator requires the decompression of its corresponding genotype, which may occur during the calculation of a mutation event, recombination event, or fitness value. Data requests on compressed genotypes propagate up the *OG* to the most recent uncompressed operations. Consequently, genotypes with higher frequencies in the population will tend to generate more data requests than low frequency genotypes, and so we can use the number of data requests as a proxy for *load*. However, there may be operations with non-zero *load* but no data requests: for instance if during time period *t* an active genotype is not mutated or if partial decompression does not propagate to both parents of a recombination event. Therefore, we maintain a boolean flag that indicates if a particular operation is required for the decompression of some active genotype, which we use in place of ‘non-zero *load*’. It is important to note that the calculation of this boolean flag requires *O(n)*, where *n* is the number of nodes which are required for the decompression of some active genotypes. Lastly, during the execution of Greedy-Load, the number of data requests for an operation may be reset (step 1) or decremented (step 2), accordingly.

To demonstrate that our approach is generally applicable to various choices of genotypes, we implemented two very different genotype models: a DNA sequence (represented by strings) and a regulatory pathway model(represented by graphs). In terms of memory allocation, a DNA sequence of length *L* occupies *L* bytes and a pathway of *k* genes occupies roughly *k*^2^ bytes. For the DNA model, we implemented four evolutionary events (that is, operations in the set): point mutations (*u*), sequence insertions (*u*_*i*_), sequence deletions (*u*_*d*_), and sequence crossover (*c*). Consequently, over the course of a simulation, the actual length of a DNA sequence may change due to insertions and deletions. To our knowledge no other SNP-based compression techniques (FREGENE or SimuPop) handl length variation.

For the pathway model, we implemented binding site loss (*u*_*l*_) and gain (*u*_*g*_), similar to the model employed in [6]. More information regarding implementation details and software can be found in the Additional file 2.

We verified the execution of the simulator using the DNA sequence genotype by comparing the input mutation and recombination rates to the estimated mutation and recombination rates inferred by the output sequences. In addition, we verified the measured sequence polymorphism and diversity using the input population, sequence length, and mutation rate. All simulations were run on a MacPro with two 2.26 GHz Quad-Core Intel Xeon processors and 16 GB 1066 MHz DDR3 memory.

## Results

To evaluate the performance of our compression algorithms—Greedy-Load and Store-Root—against the current memory management technique, Store-Active, we ran population genetic simulations under a variety of scenarios. These scenarios were chosen to test the memory and time performance of each algorithm, measured in terms of mega bytes (MB) and seconds per generation, respectively. Except for the time scaling experiments below, the time and memory usage of each simulation were recorded after an initial burn-in period, which is a standard technique employed to remove start-condition biases. We also used scaled population, generation, mutation, and recombination parameters to increase the time efficiency of the simulations [3]. The data compression ratio for a simulation is calculated as $\frac{\text{Compressed Size}}{\text{Uncompressed Size}}=\frac{k}{N}$, reported as a ratio, and the space savings is $1-\frac{k}{N}$, reported as a percentage. Thus, a Greedy-Load representation that compresses a simulation from 100 MB to 5MB has a compression ratio of 1:20 (0.05) and space savings of 95%.

### Time scaling

**Figure 2:**
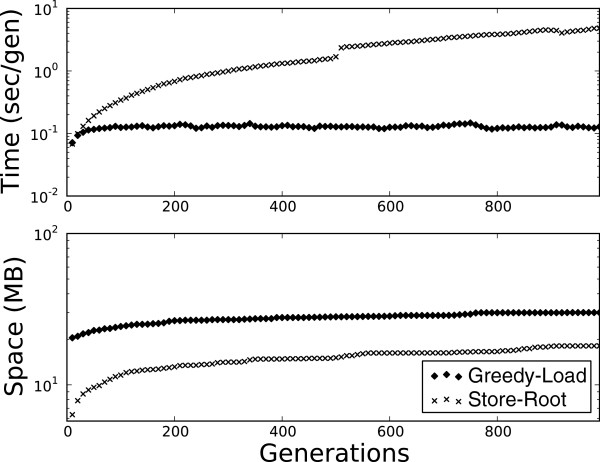
**Space and time performance of Greedy Load.** Top: The performance, in terms of time (seconds) per generation, of Greedy-Load versus Store-Root. Bottom: The performance, in terms of heap size (MB), of Greedy-Load versus Store-Root.

The goal of this work is to constrain the memory footprint of a population-genetic simulation such that as simulation time increases, memory usage remains constant, which can be trivially achieved by swapping unconstrained memory and constant time for unconstrained time and constant memory. Indeed, if decompression decisions are poor, then the latter may be the case. We measured the scaling of time (seconds per generation) as a function of simulation time over 1000 generations; results are shown in Figure 2.

For both the sequence and pathway genotypes, Store-Root exhibited log-linear (poor) scaling with respect to simulation time, whereas Greedy-Load showed constant execution time throughout the simulation. The sawtooth pattern of Greedy-Load results from the repetitive application (every *t* generations) of the algorithm.

### Parameterizing *k*, *t* in Greedy-Load

**Figure 3:**
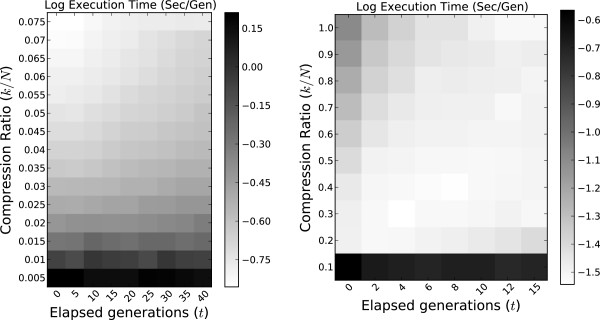
**Performance tradeoff of parameters *k* and *t*.** The tradeoff between Greedy-Load parameters *k* and *t* are presented as a heatmap of average execution times (log sec/gen), with a mutation scenario on the left and recombination on the right. Lighter colors are faster (better) simulations. The parameter *k* is given as the compression ratio (*k*/*N*), where *N*=10^3^ is constant in all the simulations.

Greedy-Load requires two parameters: *k*, the maximum number of explicitly represented genotypes (the set *c*(*v*)), and *t*, the number of elapsed generations between applications of Greedy-Load on the operation graph. Although *k* constrains the memory footprint used by the simulation, both *k* and *t* can have a combined effect on its speed, which calls for careful choice of their values. We ran multiple simulations across a dual parameter sweep of *k* and *t* under both mutation and recombination scenarios and recorded the average seconds per generation; results are shown in Figure 3.

Under a mutation-only simulation, the speed performance of Greedy-Load improves by increasing *k* and/or decreasing *t*. Except for low (<0.02) compression ratios, Greedy-Load is ‘robust’ to *k* and *t* values in that performance does not significantly degrade across the parameter space. In contrast, under simulations which employed both recombination and mutation, a linear tradeoff exists between *k* and *t*: as *k* increases *t* should increase as well. Because recombination introduces significant complexity to the OG topology — in fact, under mutation the OG is a tree — compression levels achieved by performant recombination simulations are near an order of magnitude less than the compression levels for mutation scenarios.

### Space/Time performance of policies

In this experiment, we measure the performance of each compression algorithm in terms of both time, reported as the average seconds per generation, and space, reported in MB used by the genotype heap. The memory footprint is dominated by the explicitly represented genotypes, but also counts the operations stored in the operation graph, which account for less than 0.1% of the total reported memory for all policies except Store-Root.

**Figure 4:**
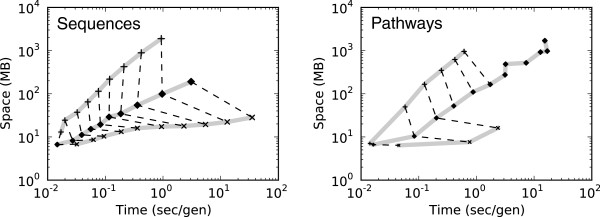
**Space and time performance of compression algorithms.** The average time per generation and memory usage required by each compression algorithm for replicate simulations of sequences (left) and pathways (right). For both time – measured in seconds per generation – and space – measured in total MB – lower values are better. The performance is measured across a range of genotype sizes: 10^5^−10^7^ bases for sequences and 100−1,000 genes for pathways. Larger genotypes require more space and longer execution times, hence a diagonal line in the space-time tradeoff. Solid lines connect a compression policy — top to bottom: Store-Active, Greedy-Load, Store-Root — and dashed lines connect genotype sizes (*e.g.,* 10^5^ nt for each policy). Greedy-Load provides 95% compression for sequences and 90% for pathways.

Time and space values were averaged over multiple simulations for both sequence and pathway genotype models. The results for both sequence and genotype models are shown in Figure 4, and depict similar performance patterns despite drastically different underlying representations.

 We compared the performance of Greedy-Load to uncompressed (Store-Active) and maximum compression (Store-Root) bounds for varying genotype sizes. As the size of the genotype increases, the space used by the simulation increases as well; however, this quantity is dependent on the level of compression. In the case of the upper bound, no compression is imposed (Store-Active). The lower bound has maximum compression — only storing one genotype, at a compression rate of 1:*N* or 1:1,000 (Store-Root). Greedy-Load provides a ‘performance knob’ between these two bounds, allowing for high levels of lossless compression without imposing significant time penalties. For the upper and lower bounds on compression, certain genotype sizes failed to complete for either space (upper bound) or time (lower bound) limitations. Sequences ranging logarithmically in size from 10^5^ to 10^7^ bp were simulated at 95% compression. Pathways ranging in size from 100 to 1,000 genes were simulated at 90% compression. 

For both genotype representations, Greedy-Load performed at competitive levels of space and time in comparison to the upper and lower bounds and completed simulations otherwise intractable to Store-Active and Store-Root.

### Greedy-Load performance in high recombination rate simulations

Recombination introduces multiple inheritance to the operation graph and so presents a unique challenge beyond a mutation-only model. Further, the rate of recombination directly relates to the amount of genotypes with multiple inheritance — or complexity of the operation graph topology. Consequently, the performance of Greedy-Load may be sensitive to the rate of recombination in a simulation.

**Figure 5:**
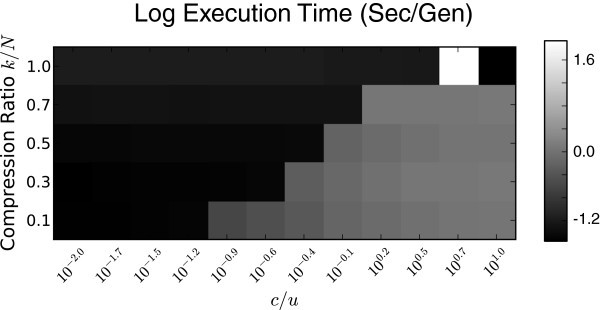
**Performance tradeoff of recombination and compression ratio.** The speed, measured in sec/gen, is plotted for Greedy-Load simulations across varying levels of compression (y-axis) and recombination (x-axis). Lighter colors are slower simulations, displayed in log-scale.

In this experiment, we measure the performance of Greedy-Load across a range of compression rates with respect to *c*/*u*, the ratio of per-base pair recombination over the mutation rate, by running a logarithmic parameter sweep of *c*/*u* from 10^−2^ to 10 (Figure 5). The mutation rate *u* is held constant at 10^−4^ and *c* is determined from the sweep parameter. The population size is 10^3^ and the sequence length is 10^4^.

Because the complexity of the operation graph increases with respect to recombination rate — moving right on the x-axis in Figure 5 — higher recombination rates require higher compression ratios (lower space savings). In fact, a phase shift exists in terms of execution time between sufficient and insufficient explicit genotypes (*k*, or compression ratio) for a given recombination rate. This decision boundary imposes limitations on the level of compression supported by Greedy-Load for high levels of recombination (*c*>>*u*). Although Greedy-Load performs correctly at any compression rate, execution time is potentially sacrificed for memory-savings.

### Imposing a memory ceiling using Greedy-Load

**Figure 6:**
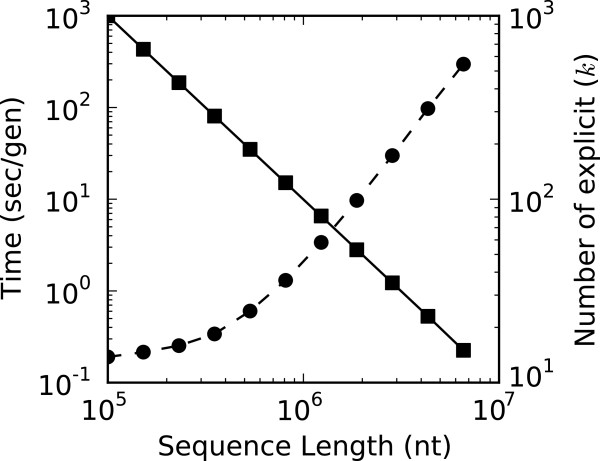
**Performance of memory constrained simulations.** We measured the ability of Greedy-Load to handle larger and larger genotypes while maintaining a memory ceiling of 100 MB. This memory ceiling was imposed by scaling the number of explicit genotypes (right axis, solid squares).

Imposing a memory ceiling constrains memory potentially at the cost of time. To investigate this tradeoff, we measured the ability of 100 MB memory-constrained simulations to handle genotypes of growing size. Sequences were scaled logarithmically from 10^5^ to 10^7^ nucleotides, where it is possible to calculate the maximum number of explicit genotypes with *k*=⌊100/(MB/genotype)⌋, with (MB/genotype) being roughly *L*/10^6^ for sequences. The execution speed for simulations under 100 MB memory constraints are shown in Figure 6, along with the maximum number of explicit genotypes, *k*, for each genotype size.

The execution time scales log-linearly with respect to the size of the data, showing that even for low *k* values, Greedy-Load performs consistently with the size of the genotype representation and does not perform arbitrarily poorly when *k* is low or genotypes are large. Although 100 MB is a threshold chosen primarily for demonstrative purposes, this experiment highlights the ability of Greedy-Load to threshold memory usage and prevent unexpected program crashes due to memory limitations.

### Simulating big data

**Figure 7:**
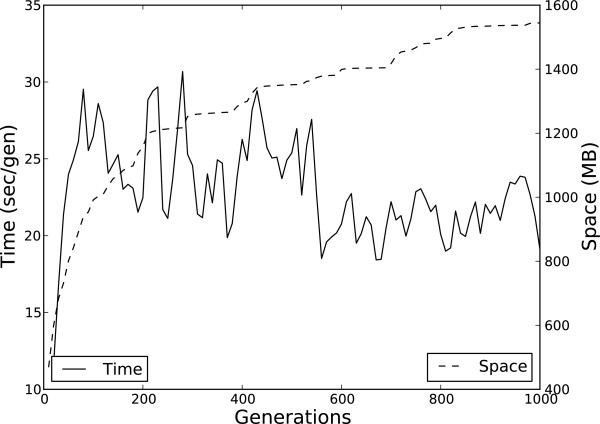
**Single-run performance of 1,000 50 Mb sequences.** The time, measured as seconds per generation, and memory heap utilized by a simulation of 1000 individuals with sequences of length 50 Mb, using 95% compression.

We simulated a population of 1000 individuals each with 50 Mb DNA sequence using base pair mutation (*u*=10^−4^), sequence insertion and deletion for 1000 generations. These parameters leveraged a scaling factor of 10^5^, so, in effect, we equivalently simulated a population of 10^8^ for 10^8^ generations with a base pair mutation rate of 10^−9^. The Greedy-Load algorithm with parameters *k*=50 (95% compression) and *t*=0 managed the compression. This simulation completed successfully, using around 1.6 GB of memory and on average 20 sec/gen (see Figure 7).

According to recent reviews, no forward-time population genetic simulator can handle this computationally demanding, yet biologically reasonable, parameter set [3,4].

## Discussion

The operation graph (OG) defined in this work presents a general and efficient data structure for lossless compression of genotypes in real-time, for the main purpose of constraining the memory footprint of forward-time population genetic simulations. By itself, the OG is capableof decreasing the memory footprint by several orders of magnitude, making possible large-scale simulations that would otherwise crash the system. However, without explicitly representing a subset of the genotypes in the OG, the time cost of decompression grows with simulation time; this amounts to trading “simulations that crash” for “simulations that never end”. Therefore, the constant-time scaling of Greedy-Load with respect to simulation time is crucial for the viability of the operation graph as a general solution. Further, the OG and Greedy-Load leverage only inheritance topology to perform compression, which means our approach is general not only to genotype representation but also to implementation details of evolutionary operations.

Recombination is an important evolutionary operation but introduces significant complexity to the operation graph: because recombination requires two parents to generate novel recombinants, decompression decisions become more complicated. For example, the path of operations used to decompress explicit genotypes become an exponentially growing dependency graph; however, Greedy-Load can successfully compress genotypes arising from recombination, although at much lower data savings in comparison to those arising from mutation-only simulations. In order to adequately handle recombination, we recorded data requests for each operation in the OG over a generation as a proxy for load, and did not calculate load explicitly. Further details on this implementation can be found in the Additional file 2.

The performance of the Greedy-Load algorithm is robust to *t*, the frequency of its execution, and *k*, the maximum number of explicit genotypes. When *k* is low, (compression rate <0.02), there was a significant drop in the time performance of Greedy-Load; otherwise, *k* and *t* had little effect on the execution time of the algorithm. In contrast, recombination benefitted from increasing *k* and *t* together. We recommend fine-tuning *k* and *t* using shorter simulations to determine which parameters to use for longer simulations.

It is important to note that although we invoke the Greedy-Load algorithm every *t* generations, other triggers may be used. For instance, the algorithm could be applied whenever |*C*(*V*)|<*k*/2, which may provide better performance when the simulator uses overlapping, instead of non-overlapping, generations. Because Greedy-Load performs accurately regardless of the value of *t*, any trigger is valid; however, the amount of topological change that the OG undergoes between applications influences the running time of the algorithm.

Because Greedy-Load diminishes the strain on the memory system while still efficiently minimizing the decompression cost of active genotypes, Greedy-Load consistently performed on-par and with less memory than Store-Active in all of our experiments. Reducing the amount of memory that is allocated and freed has a significant impact on the efficiency of the memory hierarchy. For example, reducing the overall memory overhead reduces cache misses and page faults, which, over time, has a significant impact on the speed of a simulation. So, not only does Greedy-Load constrain the memory footprint, it can do so without sacrificing speed.

Setting the maximum number of explicit genotypes reduces – not thresholds – the memory footprint of a simulation because storage of the operations is unconstrained; however, their footprint is inconsequential in comparison to explicit genotype representations. For instance, it would take 10^6^ point mutations (operations) to equal one uncompressed sequence of length 10^6^. Even in this extreme case, assuming *k*≥10, the total memory usage of the simulation would be at most a tenth more than the amount constrained by *k*. In this regard, Greedy-Load can “impose” a memory ceiling on the genotype heap.

Although constraining the memory footprint of a simulation can increase the execution time, providing a performance knob that tunes between space savings/time cost and space cost/time savings is not only a useful tradeoff but crucial for simulations with large genotypes or large populations. For example, constraining the memory footprint enables more parallel, independent simulations to run on the same node. In a recent review of forward-time simulators, sequences of length 10 Mb caused many simulators to crash [3]; in contrast, we showed that Greedy-Load could handle a population of 1000 sequences of length 10 Mb while constraining the genotype heap to 100 MB. And these benefits are not just for sequences; our compression technique also facilitated the simulation of 1,000-gene pathways while still constraining the memory to under 1 GB.

We demonstrated that our approach could be used to simulate sequences with unprecedented size; however, larger memory footprints may also manifest as more complex data structures. For instance, not only the sequence, but also its annotated features like genes or regulatory elements could be simulated as one complex system, facilitating evolutionary questions which investigate the coevolution of these integrated systems. For instance, we leveraged our compression algorithm in a recent study which investigated the neutral evolutionary trends of the *E. coli* regulatory network by simulating, at scale, the entire regulome and its underlying sequence (595 operons) over long evolutionary time scales [17]. These simulations resulted in a null distribution of system, sub-system, and operon level regulatory properties, allowing for rigorous statistical testing of neutral topological patterns. We found that the majority of *E. coli* regulatory topology — including patterns previously associated with adaptive evolution like feed-forward loops and scale free distribution — followed neutral trends.

## Conclusions

We believe our algorithm not only provides a significant advance in the computing power of population genetic simulations but also in other evolutionary simulators. These other applications may include genetic algorithms or digital genetics, which leverages complex digital organisms (computer programs) to understand evolution [18].

## Competing interests

Both authors declare that they have no competing interests.

## Authors’ contributions

TR conceived of the study, designed and implemented the algorithm, performed the simulations, and drafted the manuscript. LN conceived of the study, and participated in its design and coordination and helped to draft the manuscript. Both authors read and approved the final manuscript.

## Supplementary Material

Additional file 1 **Animation of Greedy-Load.** Visualization of Greedy-Load on *OG*s corresponding to high and low recombination scenarios Click here for file

Additionalfile 2 **Supplementary information.** Additional implementation and software details. Click here for file
