# Supplementary material for: Boosting forward-time population genetic simulators through genotype compression
Source: BMC Bioinformatics. 2013 Jun 14;14:192. doi: 10.1186/1471-2105-14-192 (PMC3700844; doi:10.1186/1471-2105-14-192)
Supplement: Additionalfile 2 — Supplementary information. Additional implementation and software details. [file 1471-2105-14-192-S2.pdf]

Boosting Forward-time Population Genetic Simulators Through Genotype Compression Supplementary Information

Troy Ruths and Luay Nakhleh

January 4, 2013

Implementation

We implemented the operation graph and compression policies in C++, which included an individual based forward-time population genetic simulator, in a library called General Purpose Population Genetic Library (GPPGLib). The code for the library can be downloaded from http://github.com/truths/GPPG. The library provides useful base classes and an example implementation of the Greedy-Load algorithm. You may use our code or develop a new implementation of the compression algorithms presented in our paper; however, we chose C++ since it is a performant language which can be wrapped and used in many other language contexts.

The operation graph and compression policies can be directly implemented as described in the paper. The only major difference between the definition of the Greedy-Load algorithm and its implementation is in the calculation of *load*. In the paper, we assumed that *load* was implicitly updated whenever a genotype was compressed or decompressed; however, because genotypes may have more than one parent, the calculation of load can become exponential. Consequently, it is not feasible to explicitly calculate load. Instead, we use *data requests* as a proxy for load.

A data request is a request for information from an operation. Over the course of a simulation, decompressing a genotype requires information from a set of operations to build the explicit representation: these data requests, which propagate up the operation graph, are recorded. These data requests

should correlate with the frequency of the genotypes in the population; however, it may be that some active genotype does not generate a data request over the course of a sampling interval (say, 10 generations). So, we not only track data requests, but an additional binary variable that stores 'true' if the operation is required by some active genotype in the population and 'false' otherwise. With these two variables, it is possible to implement the calculation of load in a time and space efficient manner.

Command Line Tool

The simulator used in this paper is implemented in standalone C++. We used CMake to allow for easy builds on all major platforms. The README file in the project directory contains build, installation, and use instructions.
